# Supplementary material for: Using experimental results of protein design to guide biomolecular energy-function development
Source: PLoS Comput Biol. 2026 Apr 22;22(4):e1014215. doi: 10.1371/journal.pcbi.1014215 (PMC13124059; doi:10.1371/journal.pcbi.1014215)
Supplement: S1 Text — (DOCX) [file pcbi.1014215.s001.docx]

**S1 Text for “Using experimental results of protein design to guide biomolecular energy-function development”**

Hugh K. Haddox, Gabriel J. Rocklin, Francis C. Motta, Devin Strickland, Samer F. Halabiya, Cameron Cordray, Hahnbeom Park, Eric Klavins, David Baker, Frank DiMaio

| **Training Parameter** | **Value** | **Description** |
| --- | --- | --- |
| Number of trees | 1000 | The number of independent decision trees trained per model. |
| Splitting criterion | Squared error | The quantity used to determine the optimal value at which to split a feature coordinate into two regions. The squared difference between the mean in each terminal node and its members (i.e., the variance). |
| Minimum samples per leaf | 20 | The minimum number of samples required in a leaf node. A split point at any depth was considered if it left at least 20 training samples in each of the left and right branches. |
| Maximum features per tree | 0.5 | The fraction of the total number of features considered when looking for the best split. At each split, a random selection of 50% of the features was considered to split. |
| Max depth | None | A maximum depth to which trees were allowed to be built. No restriction was placed on the depth of trees, instead depth was controlled implicitly using the *minimum samples per leaf* parameter. |
| Minimum impurity decrease | 0.0 | No threshold was placed on the minimum amount the squared error needed to be reduced to allow a split to occur. |
| Maximum number of leaf nodes | None | No explicit restriction was placed on the number of allowable leaf nodes. |
| Bootstrap | False | The whole training set was used to build each tree. |
| Minimum weight fraction per leaf | 0.0 | Samples were not weighted, so no minimum fraction of the total weight represented in leaf nodes was used to constrain leaves. |

**Table A in S1 Text**. Random forest regression model training parameters used for all topology-specific models. See [[1]](https://paperpile.com/c/M0f7F5/Rxir) for further details on the random forest implementation used. See <https://github.com/Haddox/design_guided_optE/tree/main/id_outlier_designs> and <https://zenodo.org/records/17330114> for data and code used to train models and identify outlier designs.


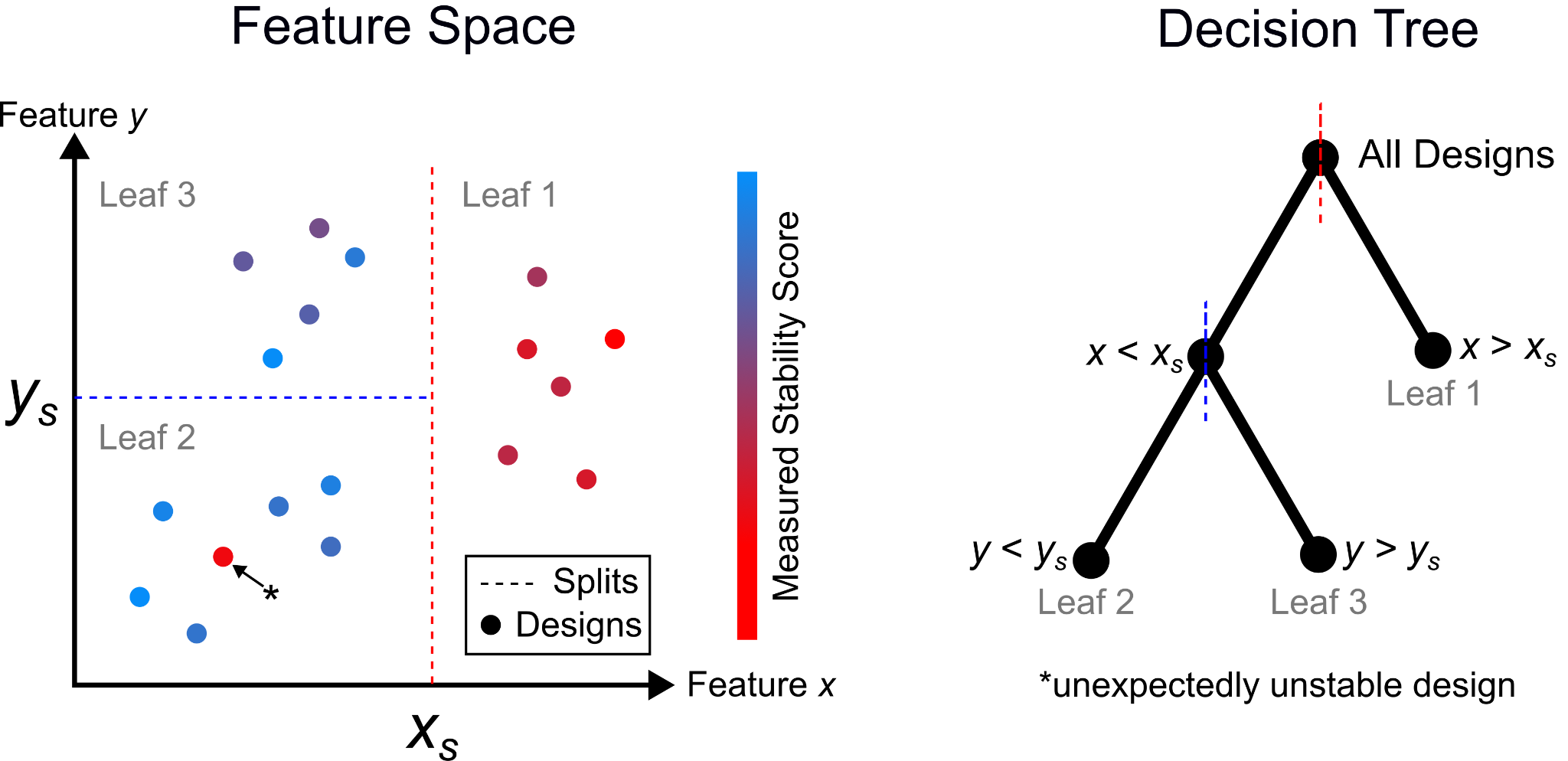


**Fig A in S1 Text. Random forest model and outlier design illustration.** Regions in a hypothetical 2-dimensional biophysical feature space determined by two splits resulting in a depth-2 decision tree with three leaves. Designs (points in feature space) are colored by cartoon experimentally measured stability scores. The indicated design with a low measured stability score (red point) in Leaf 2 will exhibit a large (negative) difference between its measure stability score and its model-predicted score since its leaf-neighbors show consistently high stability scores. Thus it may be identified as an unexpectedly unstable outlier design in this feature space.

**Random forest model training and data-driven identification of outlier designs**

We examined miniprotein designs from Rocklin et al. [[2]](https://paperpile.com/c/M0f7F5/YIDD), specifically focusing on designs from the HHH, EHEE, and EEHEE topologies. We filtered out designs with EC_50_ 95% confidence intervals that spanned more than 2 units, helping to eliminate designs with substantial uncertainty in experimentally measured stabilities. A total of 10,232 designs met these criteria (2,304 for HHH, 2,965 for EHEE, and 4,963 for EEHEE).

We fit three random forest regression models, one for each miniprotein topology (HHH, EHEE, or EEHEE), using scikit-learn [[1]](https://paperpile.com/c/M0f7F5/Rxir) (Table A in S1 Text). We trained each model to predict the experimentally measured stability scores of designs from a given topology using ~1,500 biophysical features computed for each design (the number of features varied slightly between topologies; see <https://github.com/Haddox/design_guided_optE/tree/main/id_outlier_designs> and <https://zenodo.org/records/17330114> for the full set of features used for each topology). The features are an expanded set of the ones used to train models in Rocklin et al., and include the Rosetta energy of each design. Prior to training, each feature was standardized to have a mean 0 and standard deviation 1 for designs from a given topology. Models were trained to predict experimentally measured stability scores that were generated using the unfolded state model described in Singer et al. [[3]](https://paperpile.com/c/M0f7F5/Jh2Q); these stability scores are highly correlated with the ones originally reported in Rocklin et al.

To derive a predicted stability score for each design, designs with the same miniprotein topology were divided into 50 disjoint training/predicting splits so that approximately 98% of designs were used to train a random forest model that was subsequently used to predict the stability scores of the remaining 2% of designs. Thus, each design was assigned a single predicted stability score but appeared as a training sample in 49 other random forest models.

Next, we identified outlier designs that the model predicted to have a high stability score, but actually had a low experimentally measured stability score. Specifically, we ranked designs based on the signed difference in their predicted vs. experimental stability score (= predicted - experimental), and selected the 50 designs with the most positive signed difference. Among this pool, we selected designs with trypsin and chymotrypsin EC_50_ values less than 1 in replicate 1 and 1.5 in replicate 2, an experimental stability score less than 0.3, and a predicted stability score of more than 1. These criteria produced a list of the 21 outlier designs (14 HHH, 4 EHEE, and 3 EEHEE) that we characterized using DMS. See <https://github.com/Haddox/design_guided_optE/tree/main/id_outlier_designs> for model predictions and experimental stability scores for all designs, as well as a list of all 21 outlier designs characterized by DMS.

Fig A in S1 Text illustrates at a mechanistic level how a random forest model works and how its underlying structure helped identify outlier designs. Each random forest model comprised a large collection of decision-trees trained on a point cloud of designs in *n*-dimensional space, where each point is a design and each dimension is a biophysical feature. Each decision tree begins by choosing a single feature, *x*, from a randomly selected subset of the features, along which to bisect the cloud into two subsets. The value of the *x* coordinate at which the cloud is split (call it *x_s_*) is chosen to optimally reduce a measure of disparity between the stability scores of all the designs in each half of the cloud, i.e., those designs having feature *x* values less than *x_s_* and those having *x* values greater than *x_s_*. The bisection of feature space into two regions along the *x* dimension corresponds to a splitting of the root node in a tree into two branches, and likewise a splitting of the data into two subsets–one associated to each branch. Each subset of designs may again be divided along some feature coordinate at an optimally chosen value, thus dividing feature space into a total of *k ≤ 4* regions, and resulting in a depth-2 tree with *k* leaves. The process is continued until a stopping criteria is achieved. The result is a binary tree whose terminal leaves encode for disjoint regions of the feature space and the subsets of designs that occupy each region.

The model-predicted stability score for a design, *P*, is taken to be the average stability score of all the other designs appearing in the same regions of feature space (leaf nodes) to which *P* belongs, across all decision trees in the forest. In this way, the random forest models learn the discriminating features and regions of feature space that align with the experimental stability scores, and determine a model-driven notion of proximity between designs that can be used to identify designs which are observed to be unstable but whose neighbors (members of common leaves in the random forest decision trees) are highly stable. S10 Fig shows an example of an outlier design which is unstable but is in the same leaf node of the decision tree as many highly stable designs.

**References**

1. [Pedregosa F, Varoquaux G, Gramfort A, Michel V, Thirion B, Grisel O, et al. Scikit-learn: Machine Learning in Python. Journal of Machine Learning Research. 2011;12: 2825–2830.](http://paperpile.com/b/M0f7F5/Rxir)

2. [Rocklin GJ, Chidyausiku TM, Goreshnik I, Ford A, Houliston S, Lemak A, et al. Global analysis of protein folding using massively parallel design, synthesis, and testing. Science. 2017;357: 168–175.](http://paperpile.com/b/M0f7F5/YIDD)

3. [Singer JM, Novotney S, Strickland D, Haddox HK, Leiby N, Rocklin GJ, et al. Large-scale design and refinement of stable proteins using sequence-only models. PLoS One. 2022;17: e0265020.](http://paperpile.com/b/M0f7F5/Jh2Q)
