# Supplementary figures and images for: Using experimental results of protein design to guide biomolecular energy-function development
Source: PLoS Comput Biol. 2026 Apr 22;22(4):e1014215. doi: 10.1371/journal.pcbi.1014215 (PMC13124059; doi:10.1371/journal.pcbi.1014215)

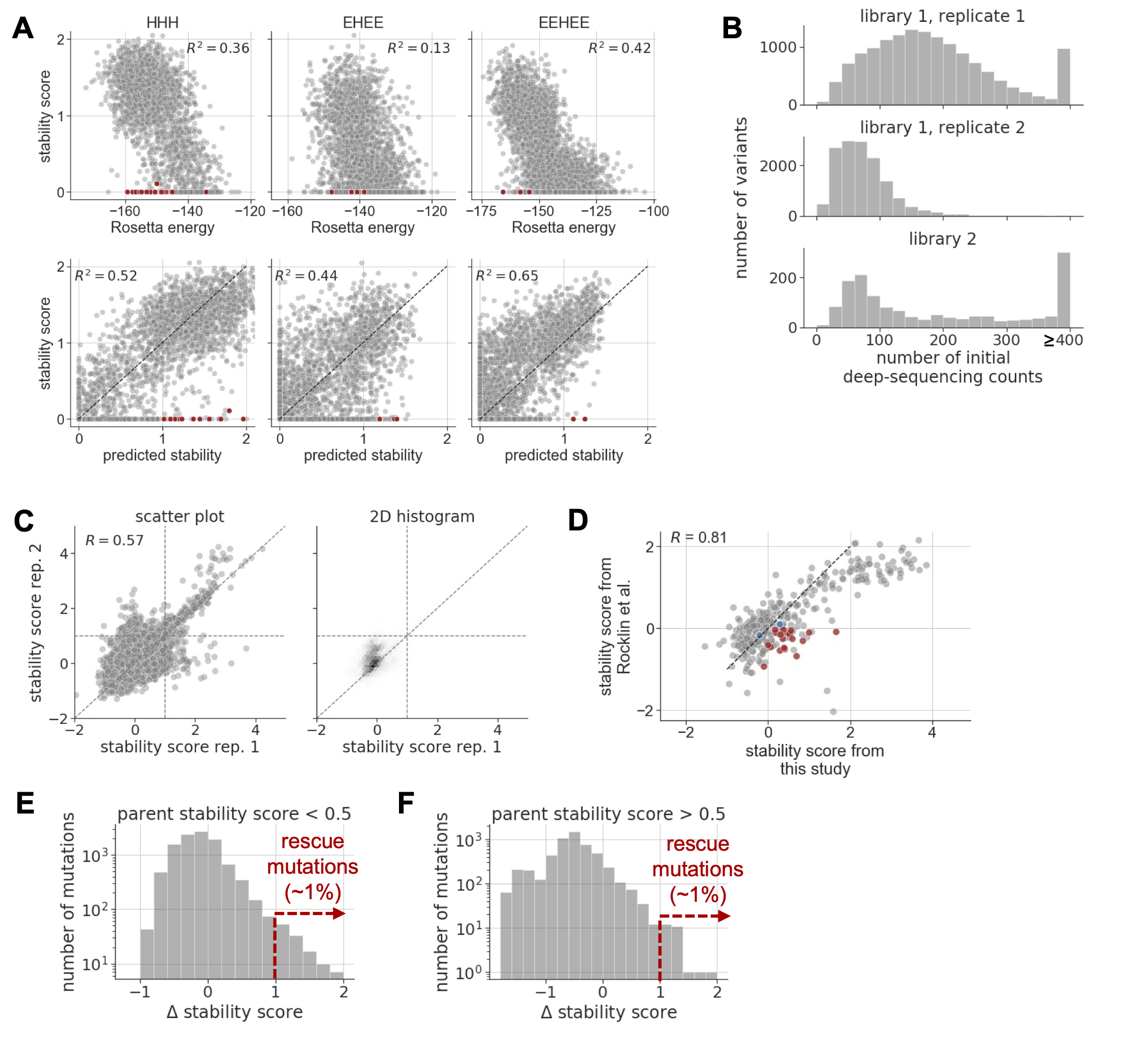

Supplement: S1 Fig — A) The top row of plots are similar to Fig 2A, but show data for all three miniprotein topologies from Rocklin et al. that we analyzed in this study (HHH, EHEE, and EEHEE; letters indicate the order of secondary-structure elements in a topology, with H = helix and E = strand). The bottom row of plots are the same as the top row, but show stabilities predicted by ML models instead of Rosetta (see Methods). Red dots show all 21 outlier designs selected for DMS, which span all three topologies. B) Histograms show the number of deep-sequencing counts of all single amino-acid variants in starting DMS libraries after transforming them into yeast cells. As described in the Methods, the 21 DMS libraries were ordered and tested as part of two larger libraries. The first of these libraries was tested in duplicate. Each plot shows data for a single replicate of a given library. Distributions are capped at 400 counts. C) Correlation of stability scores between the two biological replicates of library 1, which were generated from independent transformations of unselected plasmid libraries into yeast. In the scatter plot (left), each dot corresponds with a unique variant in the library. The 2D histogram (right) shows that most variants had low stability scores near zero in each replicate. D) Correlation of stability scores between this study and Rocklin et al. for all outlier designs, as well as 338 “ladder” miniproteins from Rocklin et al. (see Methods). Red dots show outlier designs from library 1, blue dots show outlier designs from library 2, and gray dots show ladder miniproteins. R is the Pearson correlation coefficient. The higher dynamic range of values in this study comes from experimental modifications that made the yeast surface-display scaffolding proteins less sensitive to proteolysis (see Methods). E) The same as Fig 2C, but with the y-axis scaled in log space for increased visibility of the tails of the distribution. F) The same as panel E, but now showing mutation [file pcbi.1014215.s004.tiff]

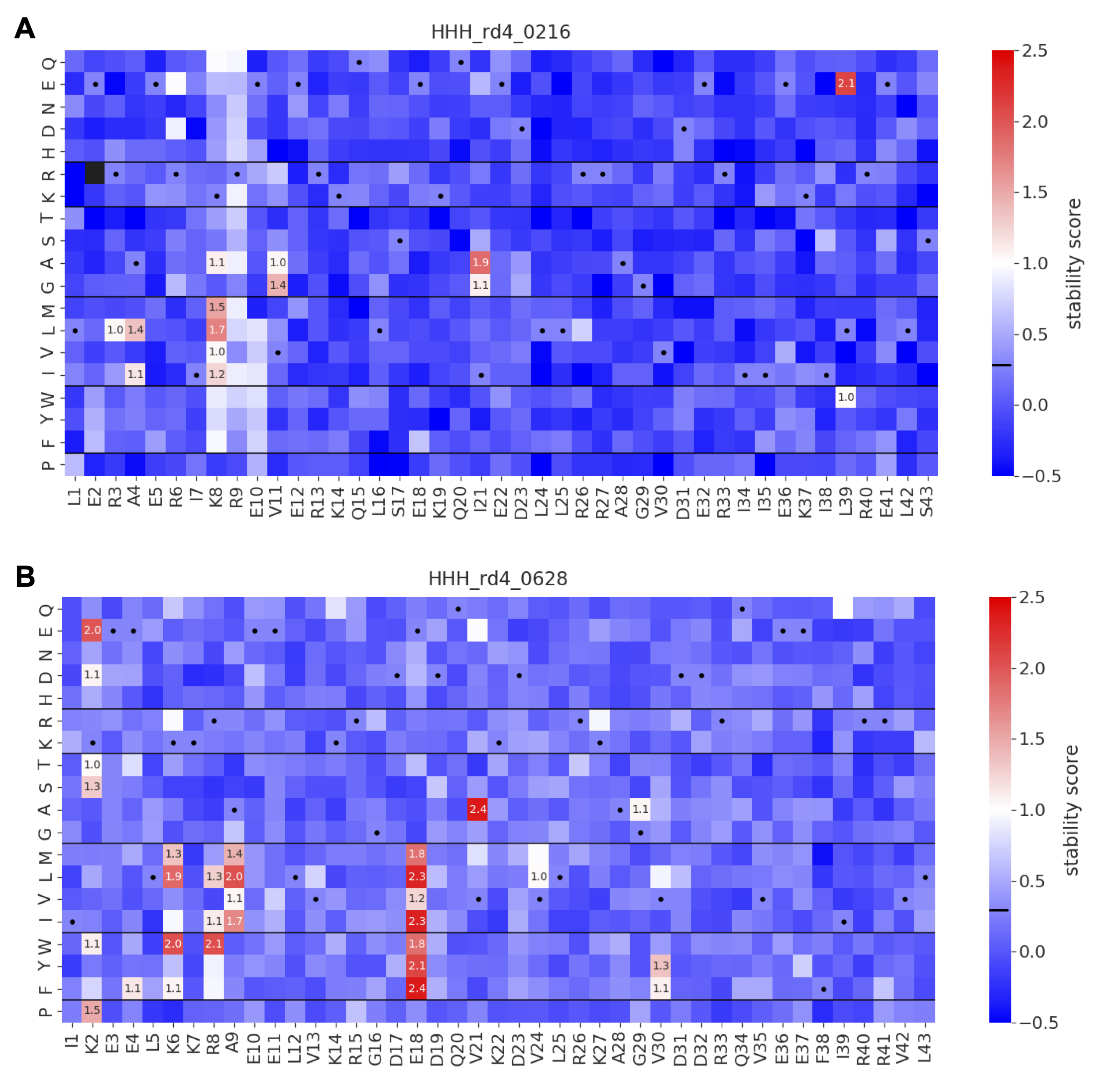

Supplement: S2 Fig — A) Data for HHH_rd4_0216 (labeled with an orange triangle in Fig 2G). B) Data for HHH_rd4_0628 (labeled with a purple circle in Fig 2G). Each box shows the stability score of a variant with a given amino-acid mutation (y-axis) at a given site (x-axis). Boxes with dots correspond to the unmutated design and show its stability score (the horizontal black line on the color bar also shows the unmutated design’s score). Numbers show stability scores for variants with scores of at least 1.0. We were unable to measure stability scores for a small number of variants (black boxes). Heatmaps of all 21 outlier designs are available at https://github.com/Haddox/design_guided_optE/tree/main/rescue_dms/heatmaps. (TIFF) [file pcbi.1014215.s005.tiff]

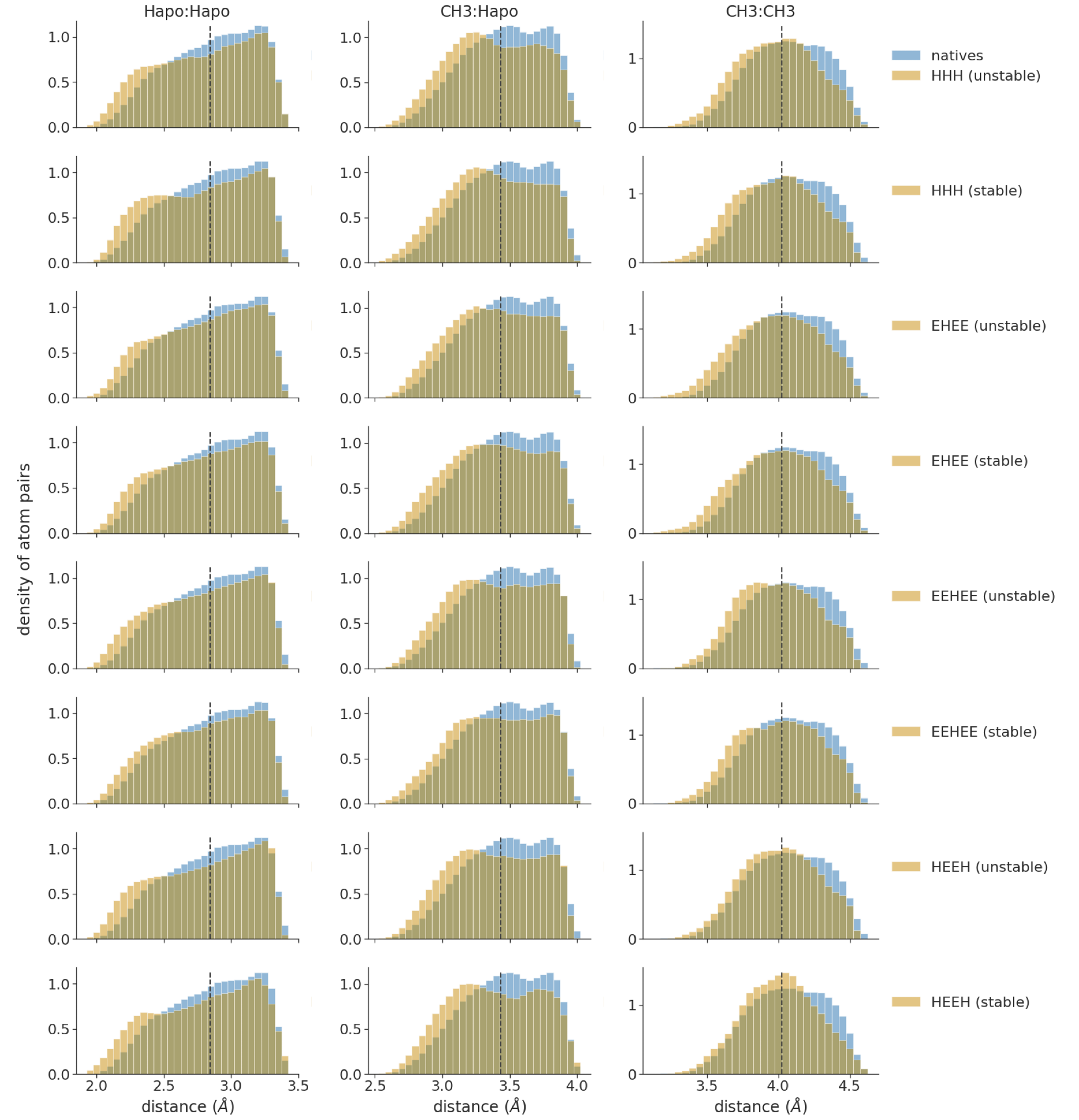

Supplement: S3 Fig — Each plot shows smoothed distributions of interatomic distances for a given atom pair (columns) within a given set of proteins (rows). Blue distributions show distances observed in 78 high-resolution crystal structures, showing data for all instances of a given atom pair within a distance cutoff of 0.5 Å greater than the sum of the radii of these atoms. Orange distributions show distances observed in a given set of designs from Rocklin et al. (from design rounds 3 and 4), with rows of plots separating designs by topology (HHH, EHEE, EEHEE, HEEH) and whether the designs were stable (stability score > 1.0) or unstable (stability score < 1.0). Vertical dashed lines show the sum of the van der Waals radii of the atom pair. Interatomic distances to the left of this line correspond to “clashes”. Columns show data for different atom pairs from hydrophobic sidechains, including pairs of hydrogens (Hapo:Hapo), hydrogens and methyl carbons (CH3:Hapo), or methyl carbons (CH3:CH3). In each plot, designs show higher levels of clashing than the crystal structures. (TIFF) [file pcbi.1014215.s006.tiff]

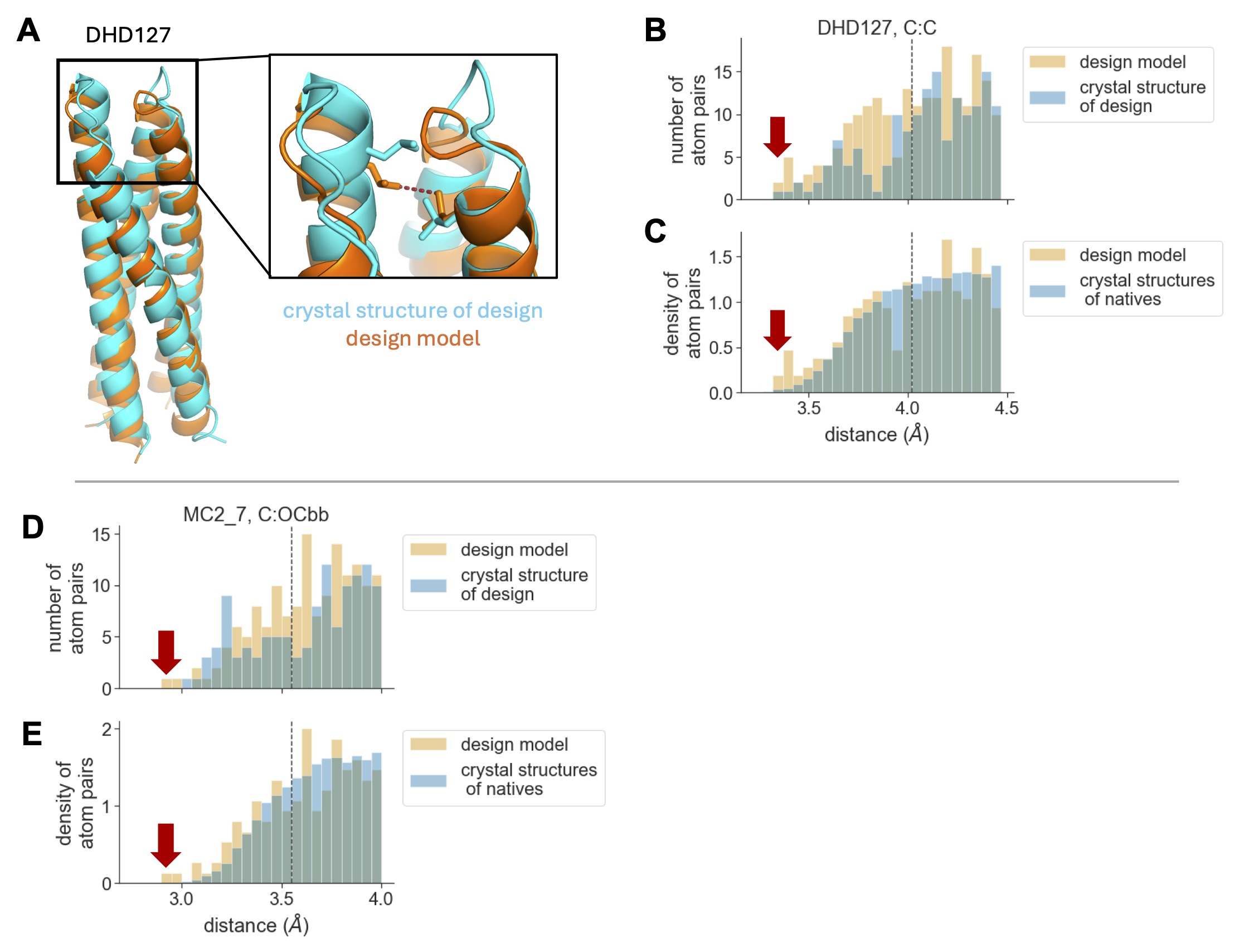

Supplement: S4 Fig — A) An example of a large clash between carbon atoms from nonpolar sidechains (see dashed red line) in a designed heterodimer (DHD127; PDB ID 6DLM). The clash is present in the design model (orange), but absent in the crystal structure (blue) due to a conformational rearrangement in the region of the clash, where one of the clashing sidechains shifts up to occupy space previously occupied by a loop. The clash is more extreme than most clashes in the design’s crystal structure and in a set of 54 crystal structures of native proteins, as shown in the next two panels. B) Histograms show the distribution of interatomic distances between all pairs of carbon atoms from nonpolar sidechains (C:C) in the DHD127 crystal structure (blue distribution) or design model (orange distribution) within a distance cutoff of 0.5 Å greater than the sum of the radii of these atoms (the dashed line shows this sum). The red arrow shows the distance of the clashing atom pair highlighted in panel A. C) Same as panel B, but the blue distribution shows interatomic distances from a set of 54 high-resolution crystal structures of native proteins. The two distributions are plotted as normalized densities to make them comparable. D and E) These three panels are similar to panels B and C, but show data for a designed NTF2 from Fig 3C, and examine clashing between pairs of carbon atoms from nonpolar sidechains and backbone oxygen atoms (C:OCbb). The red arrow shows the distance of the clashing atom pair highlighted in Fig 3C. See https://github.com/Haddox/design_guided_optE/tree/main/designs_and_xtals/compute_interatomic_distances/distance_distribution_plots for similar plots for all design-crystal pairs. (TIFF) [file pcbi.1014215.s007.tiff]

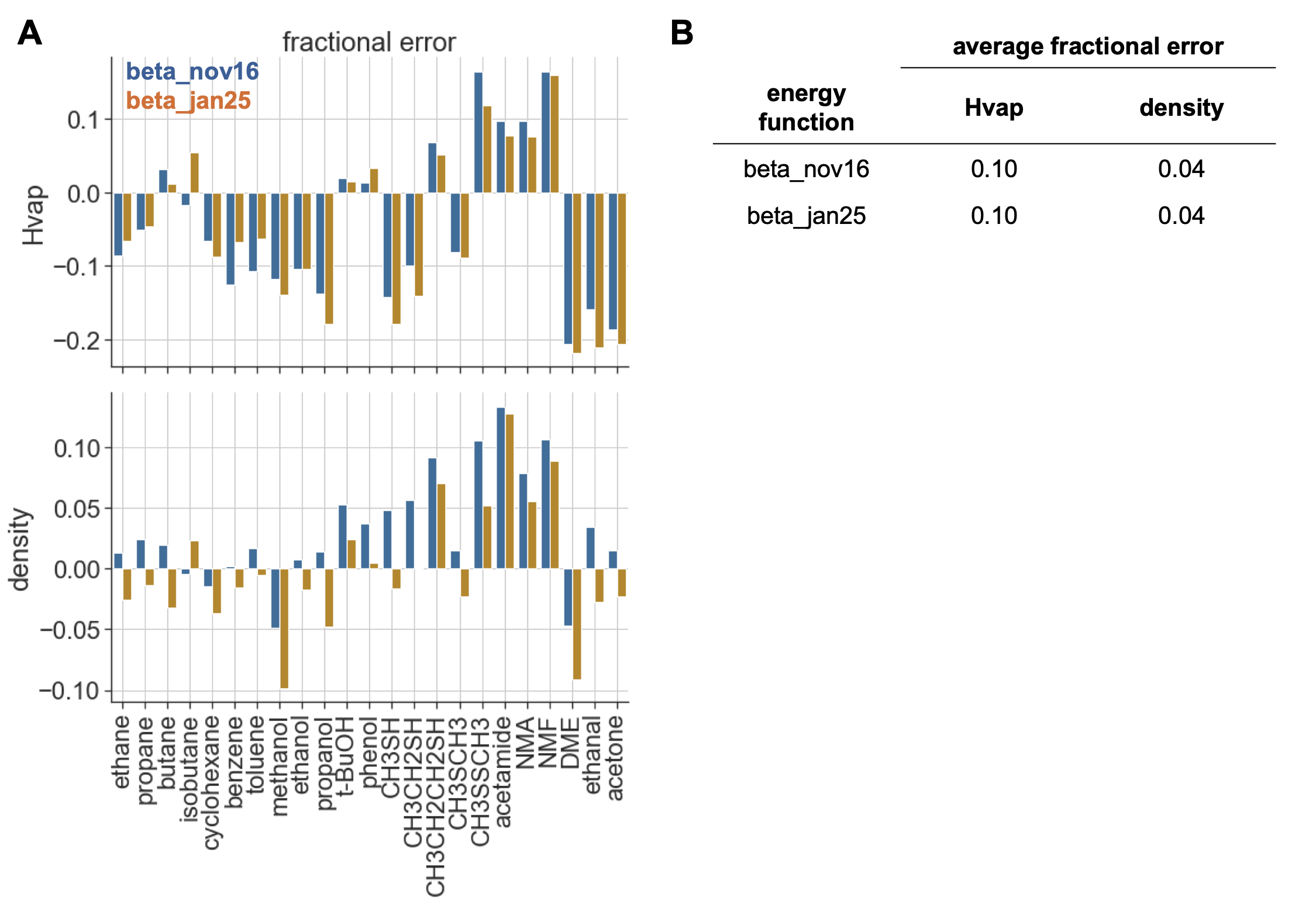

Supplement: S5 Fig — For a panel of small molecules, the benchmark uses a liquid-simulation framework to predict each molecule’s heat of vaporization and density, given an input energy function. We performed this benchmark as described in Park et al. A) Fractional error in predicted values for heat of vaporization (Hvap) and density. Each bar shows the fractional error for a given energy function (hue) on a specific small molecule (x-axis). Values are averaged over predictions from four replicate simulations for beta_nov16 or three replicate simulations for beta_jan25. (NMA = N-methylacetamide, NMF = N-methylformamide, DME = dimethyl ether). B) The average fractional error of each energy function in predicting the properties from panel A. (TIFF) [file pcbi.1014215.s008.tiff]

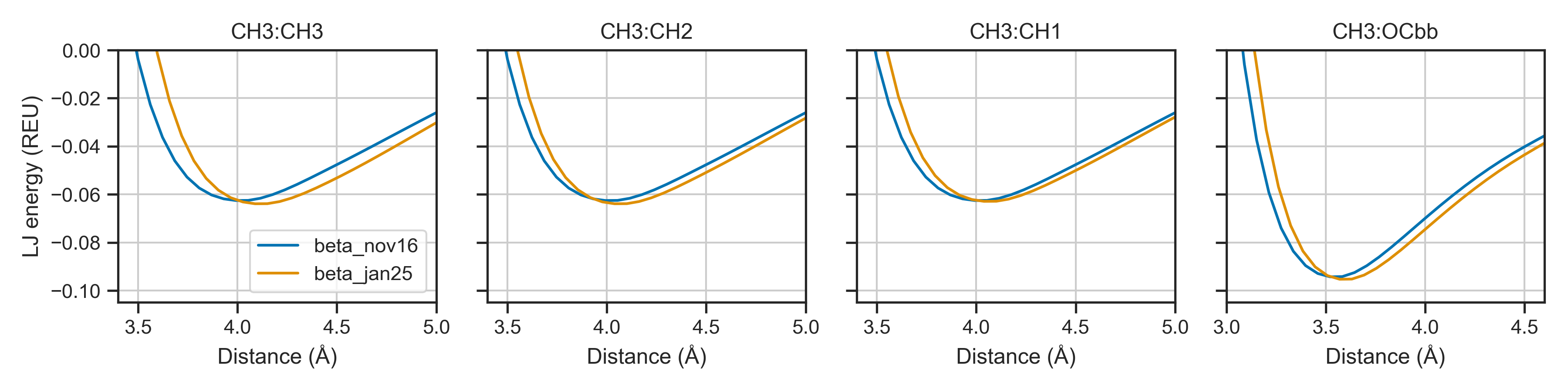

Supplement: S6 Fig — Each plot maps the LJ energy landscape for a given atom pair (see plot title) evaluated with a specific energy function (see legend). Landscapes are shifted to the right for beta_jan25 compared to beta_nov16, consistent with beta_jan25 being more repulsive at shorter distances. Shifts are subtle – on the order of tenths of Angstroms – but they are effective at reducing clashing in the distance-distribution benchmark, where overpacking is also on the order of tenths of Angstrom (Fig 4A), including for atom pairs like CH3:CH3 and CH3:OCbb for which clashing is particularly pronounced (Fig 4). The shift is most pronounced for CH3:CH3, which is expected since CH3 had the largest increase in LJ radius upon refitting (S2 Table). (TIFF) [file pcbi.1014215.s009.tiff]

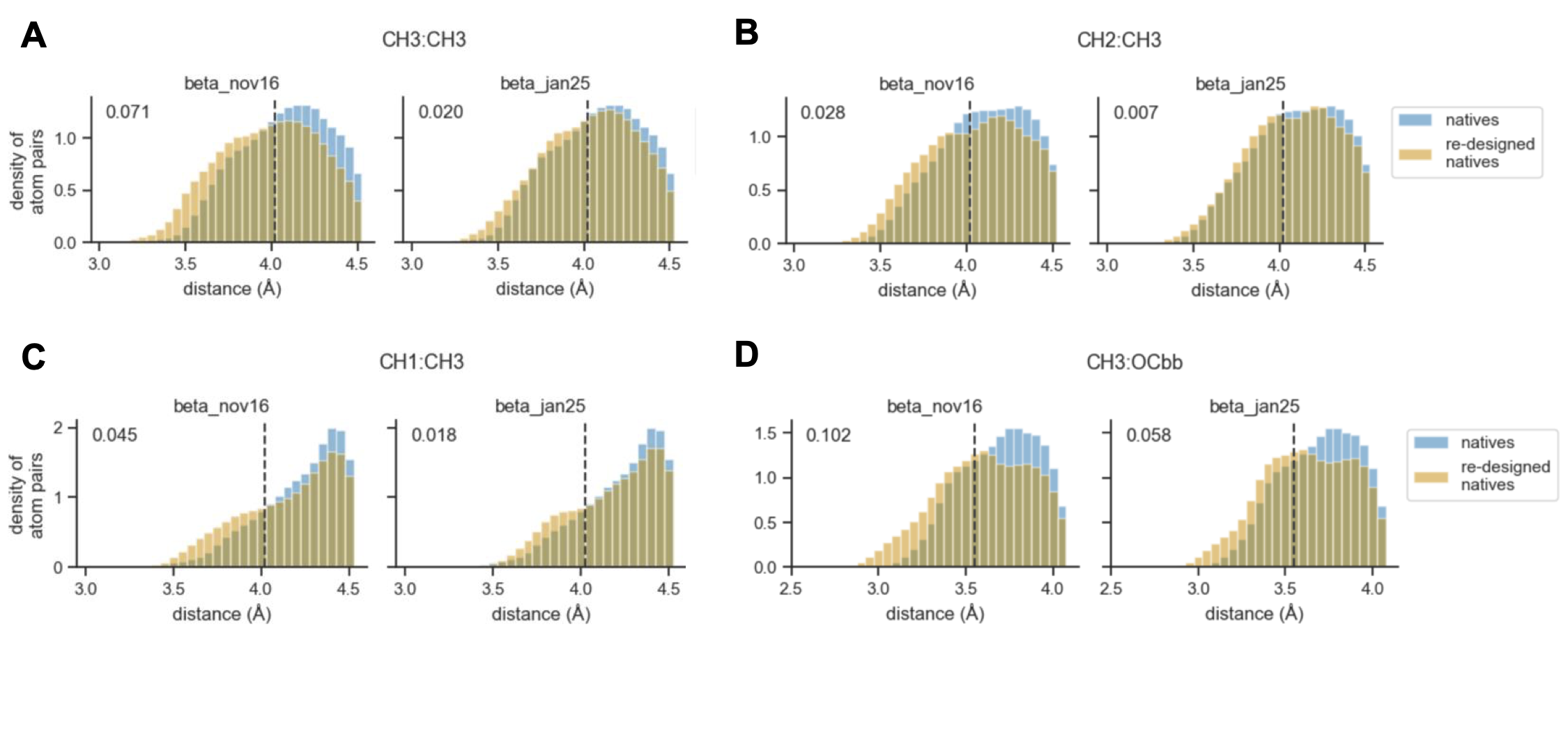

Supplement: S7 Fig — This figure is similar to Fig 4A, but shows the results of re-designing high-resolution crystal structures, rather than relaxing them. Each panel (A-D) shows data for a different atom pair (see panel titles). We performed this re-design test using 20 of the 54 crystal structures used for validation. Similar to the relax test, there is less clashing in structures re-designed with beta_jan25 compared to those re-designed using beta_nov16. For both energy functions, clashing is more pronounced in design than relax, which might be expected since design gives the energy function more freedom. There is still some residual clashing in structures re-designed using beta_jan25 (the orange distributions are still shifted to the left). (TIFF) [file pcbi.1014215.s010.tiff]

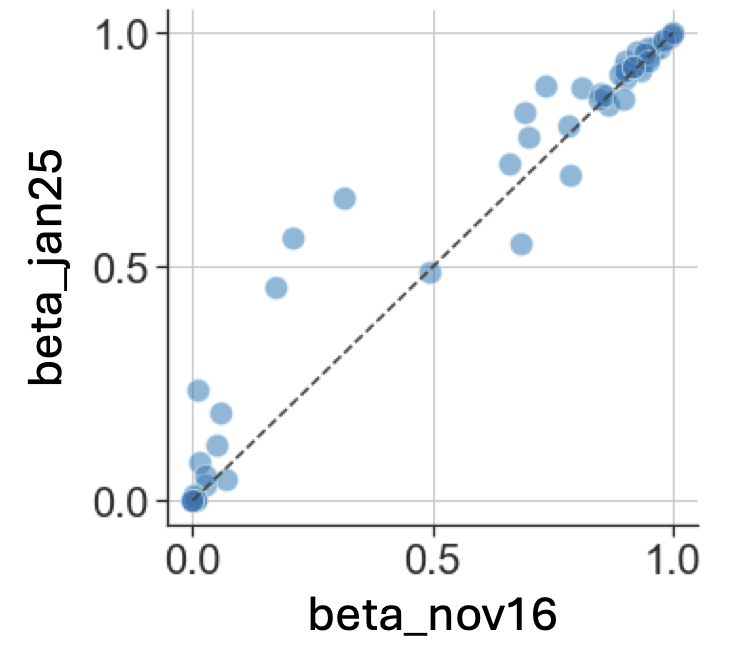

Supplement: S8 Fig — Each dot corresponds to one of the 59 crystal structures withheld from training. For each of these crystal structures, we generated a large number of decoys with non-native interfaces, relaxed the decoys with a given energy function, and then computed the Boltzmann-weighted probabilities of observing near-native structures (see Methods), with higher probabilities indicating better performance. The x and y axes report probabilities obtained by relaxing the structures with either beta_nov16 or beta_jan25, respectively. Table 1 reports the average probability for a given energy function. The higher average for beta_jan25 comes from modest increases in probabilities for several structures (dots above the diagonal line). There are only a few structures where the probability is lower for beta_jan25 (dots below the diagonal line). (TIFF) [file pcbi.1014215.s011.tiff]

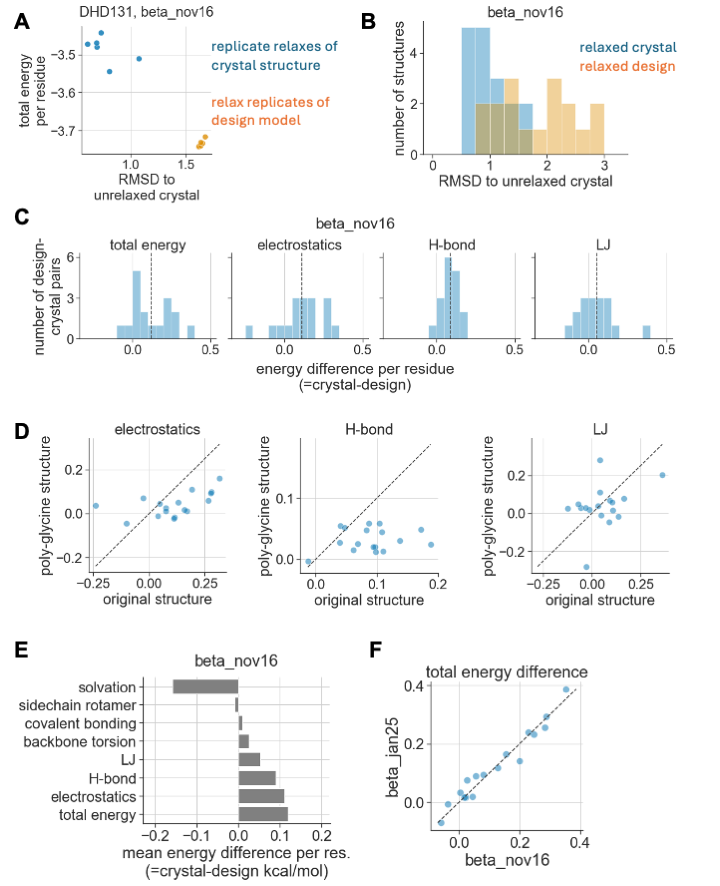

Supplement: S9 Fig — A) We separately relaxed each design model and each crystal structure, performing six independent replicates of the relax protocol. This panel shows the results for one design-crystal pair (DHD131) relaxed with the beta_nov16 energy function. Each dot corresponds the output structure from a single replicate of relaxing the crystal structure (blue dots) or the design model (orange dots). The y-axis shows each structure’s total energy per residue, while the x-axis shows each structure’s C𝛼 RMSD to the unrelaxed crystal structure. See https://github.com/Haddox/design_guided_optE/tree/main/designs_and_xtals/relax_and_scoring_protocols/energy_landscape_plots for similar plots for all design-crystal pairs. For a given pair, we selected the lowest-energy replicate from each color category to compute energy differences. B) The C𝛼 RMSD of relaxed design models (orange) and crystal structures (blue) to the unrelaxed crystal structure of the corresponding design. This plot shows data across all design-crystal pairs. C) Distributions of energy differences of design-crystal pairs. Vertical dashed lines show the mean of each distribution. D) For each relaxed design model and each relaxed crystal structure, we created a version of the structure where all side-chain atoms were replaced with a single hydrogen atom (converting the sequence to poly-glycine while keeping all backbone atoms fixed in space). This panel shows energy differences computed using the unmodified structures with all side-chain atoms intact (full pose) compared with the poly-glycine structures (poly-Gly pose), with one dot for each design-crystal pair. Many of the energy differences are closer to zero for the poly-glycine structures. E) The same as Fig 5A, but for design-crystal pairs relaxed with the beta_nov16 energy function. F) The total energy differences of design-crystal pairs are highly correlated between energy functions. (TIFF) [file pcbi.1014215.s012.tiff]
