# Supplementary material for: Using experimental results of protein design to guide biomolecular energy-function development
Source: PLoS Comput Biol. 2026 Apr 22;22(4):e1014215. doi: 10.1371/journal.pcbi.1014215 (PMC13124059; doi:10.1371/journal.pcbi.1014215)
Supplement: Attachment — Submitted filename: response_letter.pdf [file pcbi.1014215.s013.pdf]

UNIVERSITY of WASHINGTON
DEPARTMENT OF BIOCHEMISTRY
School of Medicine

Frank DiMaio
Department of Biochemistry
University of Washington
Box 357350
Seattle, WA 98195
(206) 221-5283
dimaio@u.washington.edu

Mar 23, 2026

PLoS Computational Biology

Dear Dr. Chen and Dr. Elofsson,

We thank the reviewers for their thoughtful and constructive suggestions. We have made the following major changes to the manuscript.

Testing for underpacking in design. One of the reviewers raised the concern that the new energy function might result in *underpacking* during design, given its LJ potential is more repulsive. To test this concern, we re-designed high-resolution crystal structures of native proteins with both the new energy function (beta_jan25) and its predecessor (beta_nov16), and then examined atomic packing in the resulting designs.

We did not see underpacking in designs made by beta_jan25. These results are shown in a newly added supplemental figure (S7 Fig). As expected, designs made using beta_nov16 had clashes. Designs made using beta_jan25 had reduced levels of clashing. However, they still had higher levels of clashing than the crystal structures used as input for design (S7 Fig; the orange distributions are still shifted to the left). Thus, the results point to residual levels of overpacking, rather than underpacking.

Discussion of alternative functional forms. Given there are still residual levels of overpacking in proteins relaxed (and designed) using the new energy function. One reviewer suggested we discuss alternative functional forms to model van der Waals interactions. We now do so in the following paragraph we added to the Discussion:

> *Despite this improvement, the problem persists at a low level for some atom pairs. Future work could examine ways to completely eliminate the problem. In this paper, we focused on refitting a small number of parameters in context of the 12-6 LJ potential. But, future work could explore more dramatic refitting schemes, including alternative functional forms for modeling van der Waals interactions [35].*

Below are point-by-point responses to each reviewer comment. The reviews are in **blue** and our responses are in **black**.

Thanks for your time and consideration,

Frank DiMaio

Frank DiMaio

Reviewer #1: The authors performed deep mutational scanning on mini-proteins designed using the Rosetta energy function, and identified "rescue" mutations that convert unstable designs into stable proteins. Through analysis of these mutations, they uncovered common "failure modes" of the energy function. Among several such modes, they focused on one involving steric clashes or overpacking in the hydrophobic core. Notably, similar overpacking artifacts were observed when applying Rosetta to refine high-resolution crystal structures. To address this, the authors reparameterized a small subset of energy function terms to better recapitulate native-like atomic contact distances. They demonstrate that the revised energy function corrects the steric clashes or overpacking in structure refinement tasks, while maintaining comparable performance on benchmarks for predicting ΔΔG values of monomer and interface mutations.

Given the widespread use of the Rosetta energy function, this study is highly relevant and delivers a practically useful update to an important tool. Nevertheless, I have the following concerns that need to be addressed before publication.

While overpacking appears as one failure mode among others in the deep mutational scanning data, it seems to represent only a minor subset of the overall design failures. Although it is reasonable to focus on this problem at first, it is essential to evaluate whether the proposed reparameterization disrupts the delicate balance between different energy terms in the original function. Such rebalancing could inadvertently exacerbate other known issues or introduce new ones.

Currently, the authors support their revision primarily by showing subtle improvements in C–H and H–H contact distance distributions during high-resolution refinement tasks. However, in benchmark tests assessing correlation with experimental ΔΔG measurements, the performance of the revised model is indistinguishable from that of the original. Taken together, these results are insufficient to fully rule out unintended consequences of the parameter changes, especially the balancing between van der Waals (VDW) packing term and other energetic components.

To provide a more comprehensive evaluation, the revised energy function should be applied to the original set of designed mini-proteins to compare its predictions directly against the deep mutational scanning data. Specifically, it should be assessed how well the updated model predicts various classes of rescue mutations, which should include not only favorable large-to-small substitutions (which alleviate overpacking), but also small-to-large mutations. This would help determine whether the refitting disproportionately favors certain mutation types or leads to overprediction of destabilizing large-to-small changes as beneficial.

Thank you for the thoughtful comment. We agree that it is important to evaluate whether the reparameterization disrupts the balance between terms in the energy function.

The reviewer suggests evaluating the ability of the new energy function to predict rescue mutations in the DMS data. We have performed this analysis, and the below figure shows the results for large-to-small (left plot) and small-to-large (right plot) rescue mutations. Each plot compares mutational effects predicted by the beta_nov16 energy function (x axis) to those predicted by the new beta_jan25 energy function (y axis).

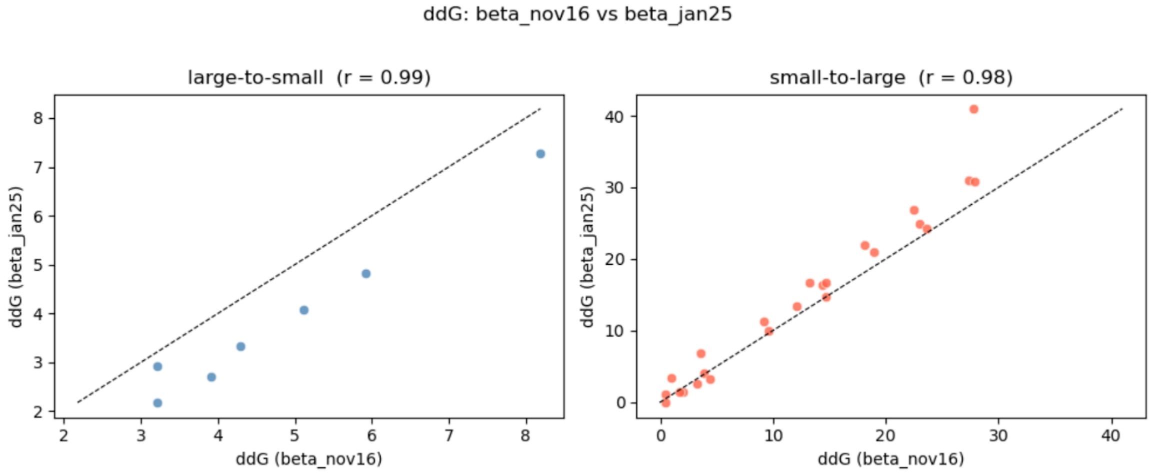

In general, the predictions are correlated between energy functions, and both energy functions predict that most rescue mutations are deleterious (ddG > 0). Large-to-small mutations are consistently predicted to be more deleterious by beta_nov16, consistent with this energy function being more tolerant of the originally designed clash. In contrast, small-to-large mutations are consistently predicted to be more deleterious by beta_jan25, consistent with this energy function being less tolerant to clashes that are expected to arise from such mutations without extensive conformational sampling. Predictions were obtained through the standard Rosetta protocol for estimating the ddG of mutations, which involves limited sampling.

We decided not to include these results in the manuscript for two main reasons. First, the stabilized designs with rescue mutations lack experimental structures. We have computational models of the original unmutated designs. But, computational models often have Angstrom-level differences from design models. And some rescue mutations would presumably require large structural rearrangements to be accommodated. This is especially true for the small-to-large mutations, which often occurred in the protein core, where increases in sidechain size would cause dramatic clashes in the designed conformation. Despite recent advances in structure prediction, we think it unlikely that we could predict mutant structures accurately enough to evaluate the energy function. Second, there are only a few dozen small-to-large or large-to-small rescue mutations to use for evaluating the energy function. In contrast, the ddG benchmarks that we include in the manuscript involve evaluating the energy function using hundreds of mutations made to proteins with high-resolution crystal structures.

In our original submission, our main approach to test for imbalances in the new energy function was to evaluate it on the same array of biophysical benchmarks originally used to evaluate beta_nov16. The new energy function did not perform worse on any of the benchmarks, and it performed better on benchmarks related to atomic packing and predicting protein interfaces (Table 1). Thus, these benchmarks do not detect obvious imbalances in the new energy function. Ultimately, we agree we cannot fully rule out the possibility that our changes introduced imbalances not detected by the benchmarks, but the completeness of the benchmarks suggests they likely do not.

Reviewer #2: A common goal for the field of protein design is to learn from failed and successful design campaigns so that future attempts have higher success rates. This is

an interesting study that uses site saturation mutagenesis of large numbers of de novo designed proteins to learn what went right and wrong during design. The aim was to determine if the energy function used during the design process has systematic pathologies that prevent choosing the most favorable amino acid at each sequence position. Primary findings include the observation that the designed proteins can most often be stabilized by mutating a partially buried polar amino acid to a non-polar amino acid, and that the energy function failed to detect destabilizing clashes at a small set of sequence positions. The second observation was used to guide parameterization of the Rosetta Lennard-Jones potential. This paper will be of general interest to the protein design field. Minor revisions that address the following questions are recommended.

1) The differences made to the LJ potential (table S2) appear to be very small. It would be helpful to include some figures that plot the new potential over the previous potential (with all weights applied).

Thanks for the suggestion. We have added a new supplemental figure (S6 Fig) that shows the difference in the LJ potential between energy functions for atom pairs that show the clashing problem. The potential is shifted to the right for the refit energy function (beta_jan25), consistent with this energy function being more repulsive at shorter distances. Shifts are subtle – on the order of tenths of Angstroms – but they are effective at reducing clashing in the distance-distribution benchmark, where overpacking is also on the order of tenths of Angstrom (Fig 4A), including for atom pairs like CH3:CH3 and CH3:OCbb for which clashing is particularly pronounced (Fig 4).

2) One concern when increasing repulsive forces is that it will lead to underpacking at some residue positions during design. What evidence is there that the changes implemented here do not lead to underpacking?

We appreciate the concern. At the top of our response letter, we describe how we added a new supplemental figure that tests for this concern. We did not find signs of underpacking in design.

Reviewer #3: This manuscript presents a novel and systematic approach for improving biomolecular energy functions by learning from the failures of de novo protein designs. The following minor issues should be addressed to enhance clarity:
1. Lines 150–151 state: “We also performed biological replicates of the entire experiment for 19 of the 21 outlier designs.” Could the authors clarify why biological replicates were carried out for only 19 of the 21 outlier designs?

We ordered DMS libraries in two separate oligonucleotide pools. The first pool had libraries for 19 outlier designs. The pool had libraries for the 2 other outlier designs. We processed each pool separately, performing duplicate DMS experiments on the first pool and a single DMS experiment on the second pool. We describe these details in the *Methods* section. And we have now updated the *Results* section to briefly describe this:

> *We also performed biological replicates of the entire experiment for 19 of the 21 outlier designs (the 2 other outlier designs were tested separately with a single replicate experiment; see Methods)*

2. Lines 343–344 state: “Following optimization with dualoptE, we manually fine-tuned

parameters to further improve atom-pair distribution fits." Could the authors briefly explain the procedure for manual parameter fine-tuning?

We have added a brief explanation to the *Methods* section:

> *After running the automated dualoptE protocol, we manually fine tuned parameters to further improve performance on the distance-distribution benchmark used in training. That is because some atom pairs still showed low residual levels of clashing after the dualoptE protocol. We found that manually increasing radii by a small amount helped reduce this residual clashing, slightly increasing performance on the atom-pair distribution benchmark without decreasing performance in the benchmarks. The residual clashing after dualoptE may have been because signal of this residual is weak in the benchmark's loss function when summed over all atom pairs.*

3. The study focuses primarily on retraining the beta_jan25 energy function. Would it be more appropriate to present data for beta_jan25 rather than beta_nov16 in Figure 5A?

Thanks for the suggestion. We have updated Figure 5A to show data for beta_jan25.

4. Some steric-clashing issues may not be fully resolved by the dualoptE protocol and the Lennard-Jones potential. Could alternative functional forms or protocols be considered? The authors may wish to briefly discuss this possibility.

Good suggestion. We have now added a paragraph to the Discussion on how future work could consider alternative functional forms, as we describe in more detail at the top of our response letter.
